# Supplementary material for: ASPEN, a methodology for reconstructing protein evolution with improved accuracy using ensemble models
Source: eLife. 2019 Oct 17;8:e47676. doi: 10.7554/eLife.47676 (PMC6797483; doi:10.7554/eLife.47676)
Supplement: Transparent reporting form [file elife-47676-transrepform.docx]

***eLife’s* transparent reporting form**

We encourage authors to provide detailed information *within their submission* to facilitate the interpretation and replication of experiments. Authors can upload supporting documentation to indicate the use of appropriate reporting guidelines for health-related research (see [EQUATOR Network](http://www.equator-network.org/%20)), life science research (see the [BioSharing Information Resource](https://biosharing.org/)), or the [ARRIVE guidelines](http://www.plosbiology.org/article/info:doi/10.1371/journal.pbio.1000412) for reporting work involving animal research. Where applicable, authors should refer to any relevant reporting standards documents in this form.

If you have any questions, please consult our Journal Policies and/or contact us: [editorial@elifesciences.org](mailto:editorial@elifesciences.org).

**Sample-size estimation**

- You should state whether an appropriate sample size was computed when the study was being designed
- You should state the statistical method of sample size computation and any required assumptions
- If no explicit power analysis was used, you should describe how you decided what sample (replicate) size (number) to use

Please outline where this information can be found within the submission (e.g., sections or figure legends), or explain why this information doesn’t apply to your submission:

Since the bulk of analysis presented in this manuscript is based on simulated sequence data, and no inferences about a population were made based on a sample from that population, quantitative sample-size estimation was not necessary. The choices of orthogroup subsample size (90% and 50%) are discussed in the last paragraph of subsubsection “Framework for generating an ensemble of topologies and quantifying uncertainty” of the “Quantifying reconstruction uncertainty with sequence subsampling” subsection of the “Results” section. The choices of number of subsamples drawn (50 90% subsamples, 1000 50% subsamples) were a compromise between saturation and computational time considerations.

**Replicates**

- You should report how often each experiment was performed
- You should include a definition of biological versus technical replication
- The data obtained should be provided and sufficient information should be provided to indicate the number of independent biological and/or technical replicates
- If you encountered any outliers, you should describe how these were handled
- Criteria for exclusion/inclusion of data should be clearly stated
- High-throughput sequence data should be uploaded before submission, with a private link for reviewers provided (these are available from both GEO and ArrayExpress)

Please outline where this information can be found within the submission (e.g., sections or figure legends), or explain why this information doesn’t apply to your submission:

To ensure broad applicability we generated 600 synthetic datasets, allowing us also to push the trees into different degrees of difficulty. In our case, there is no explicit biological vs. technical replication, but one may consider each of the 600 synthetic datasets as a biological replicate and the thousands of subsampled trees produced within that the technical replication we capitalized on to improve our estimation of the biological problem. No data was excluded from study. The scope and preparation of the protein sequence data set is addressed in the “Defining protein families for testing” subsubsection of the “Quantifying reconstruction uncertainty with sequence subsampling” subsection of the “Results” section, as well as the “Sequences” subsection of the “Materials and Methods” section. The latter section specifies access to all synthetic sequences and materials pertaining to their simulation (DOI: 10.6084/m9.figshare.5263885) and natural sequences from the LacI transcription factor family (included in supplementary material).

**Statistical reporting**

- Statistical analysis methods should be described and justified
- Raw data should be presented in figures whenever informative to do so (typically when N per group is less than 10)
- For each experiment, you should identify the statistical tests used, exact values of N, definitions of center, methods of multiple test correction, and dispersion and precision measures (e.g., mean, median, SD, SEM, confidence intervals; and, for the major substantive results, a measure of effect size (e.g., Pearson's r, Cohen's d)
- Report exact p-values wherever possible alongside the summary statistics and 95% confidence intervals. These should be reported for all key questions and not only when the p-value is less than 0.05.

Please outline where this information can be found within the submission (e.g., sections or figure legends), or explain why this information doesn’t apply to your submission:

Given the size of the presented simulation study, raw results could not be practically presented. Custom statistics defined for our study (accuracy, precision) are presented in the subsubsection “Framework for generating an ensemble of topologies and quantifying uncertainty” of the “Quantifying reconstruction uncertainty with sequence subsampling” subsection of the “Results” section. Figure 1E shows box plots with boxes encompassing the 25^th^ through 75^th^ quantiles, whiskers encompassing the 95% confidence intervals, and outliers shown individually. Figure 4 shows accuracy averages over protein families binned according to precision, as described in the figure caption.

(For large datasets, or papers with a very large number of statistical tests, you may upload a single table file with tests, Ns, etc., with reference to sections in the manuscript.)

**Group allocation**

- Indicate how samples were allocated into experimental groups (in the case of clinical studies, please specify allocation to treatment method); if randomization was used, please also state if restricted randomization was applied
- Indicate if masking was used during group allocation, data collection and/or data analysis

Please outline where this information can be found within the submission (e.g., sections or figure legends), or explain why this information doesn’t apply to your submission:

Group allocation was not performed in our study, as all protein families underwent the same analysis procedure.

**Additional data files (“source data”)**

- We encourage you to upload relevant additional data files, such as numerical data that are represented as a graph in a figure, or as a summary table
- Where provided, these should be in the most useful format, and they can be uploaded as “Source data” files linked to a main figure or table
- Include model definition files including the full list of parameters used
- Include code used for data analysis (e.g., R, MatLab)
- Avoid stating that data files are “available upon request”

Please indicate the figures or tables for which source data files have been provided:

All input sequences are provided for simulated (DOI: 10.6084/m9.figshare.5263885) and LacI (supplementary material) protein families. Code used to perform ASPEN topology reconstruction is available at https://github.com/NaegleLab/ASPEN
